# Hymenolepiasis in a Pregnant Woman: A Case Report of Hymenolepis nana Infection

**DOI:** 10.7759/cureus.3810

**Published:** 2019-01-01

**Authors:** Venkataramana Kandi, Sri Sandhya Koka, Mohan Rao Bhoomigari

**Affiliations:** 1 Microbiology, Prathima Institute of Medical Sciences, Karimnagar, IND

**Keywords:** hymenolepiasis, hymenolepis nana, h. nana, h. diminuta, children, adults, asymptomatic, pregnant woman

## Abstract

Hymenolepiasis is an infection caused by *Hymenolepis nana* (*H. nana*) and *H. diminuta* (*H. diminuta*). Hymenolepiasis is prevalent throughout the world with human infections with *H. nana* being frequently reported in the literature as compared to *H. diminuta*. Hymenolepiasis is more frequent among children, and most human infections remain asymptomatic and self-limited. Symptoms including abdominal pain, diarrhea, and vomiting are frequently noted in the cases of heavy infections. We report a case of hymenolepiasis caused by *H. nana* in a pregnant woman.

## Introduction

Human infection caused by the cestodes belonging to the genus *Hymenolepis *is called as hymenolepiasis. The cestodes are broadly classified as pseudophyllidean and cyclophyllidean cestodes. *Hymenolepis *species (spp.) fall into the cyclophyllidean group, which is characterized by the presence of four cup-like structures in the scolex/head called as suckers. The suckers are either armed (presence of hook-like structures) or unarmed (no hooks). *Hymenolepis *spp. are armed with the presence of a single round of hooks around the suckers. Among the *Hymenolepis *spp., *H*. *nana *is commonly called as a dwarf tapeworm and *H*. *diminuta *is referred to as a rat tapeworm. *H*. *nana *frequently causes human infections and may also cause infections in rats, whereas *H*. *diminuta *exclusively causes infections in rat and rarely infects humans. Man is the definitive host for *H*. *nana *and both man and rat act as definitive hosts for *H*. *diminuta*. *H*. *nana *var. *fraterna *is a genetic variant of *H*. *nana *for which rat can also act as a definitive host. The arthropod insects including beetles (*Tenebrio molitor*:* *mealworm beetle, *Tribolium castaneum*:* *flour beetle) and fleas (*Ctenocephalides*:* *cat flea, dog flea; *Xenopsylla cheopis*: rat flea; *Pulex irritans*:* *human flea) ingest the eggs present in the feces/environment and act as intermediate hosts, in whom the encysted larvae exist [1].

Most human infections of *Hymenolepis *spp. remain asymptomatic and only cause self-limited infections, where the parasite is eliminated naturally. Few infections, in most instances as a result of heavy infestation, may show typical clinical symptoms that include abdominal pain, vomiting, and diarrhea. Pregnant women are a special group of individuals who could be more susceptible to infections and warrant special attention and care. Infections during pregnancy may affect the pregnancy as such and the neonatal health. Also, the therapeutic interventions during pregnancy may result in unexpected complications to both maternal and child’s health [2]. Previous research has also highlighted the fact that neonates exposed to maternal infections in utero are at an increased risk of developing allergy/inflammatory and metabolic disorders [3]. We present a case of *H*. *nana *infection in a pregnant woman in her 35^th^ week of gestation and presented with typical symptoms.

## Case presentation

**Figure 1:**
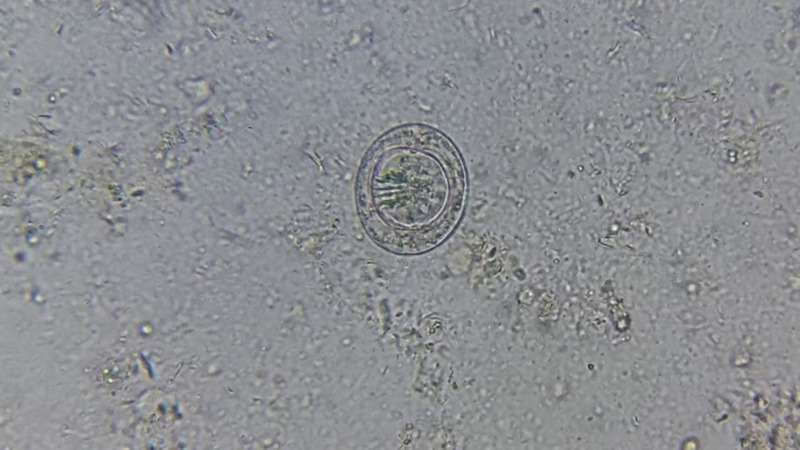
Direct stool wet mount showing non-bile stained egg of Hymenolepis nana

A 24-year-old female patient in her 35^th^ week of gestation presented to the medical out-patient department with complaints of loose stools not associated with blood, with a frequency of 10 times a day for the past two days. The patient gave a history of two episodes of vomiting and pain in the abdomen. There was no history of fever, rash, burning micturition, white discharge, and vaginal bleeding. The first and second trimesters were uneventful. The patient gave a history of appendectomy five years back. General physical examination was normal. Complete blood picture revealed mild anemia (hemoglobin: 9.5 g%) and there was no eosinophilia. Ultrasound revealed single live intrauterine fetus (SLIUF) in cephalic presentation with a mean gestational age of 35 to 36 weeks and mild polyhydramnios. Stool examination for intestinal parasites and for the presence of occult blood was advised. Direct stool examination using a simple wet mount with saline and iodine mount revealed eggs/ova that morphologically resembled *H*. *nana, *as shown in Figure 1.

**Figure 2:**
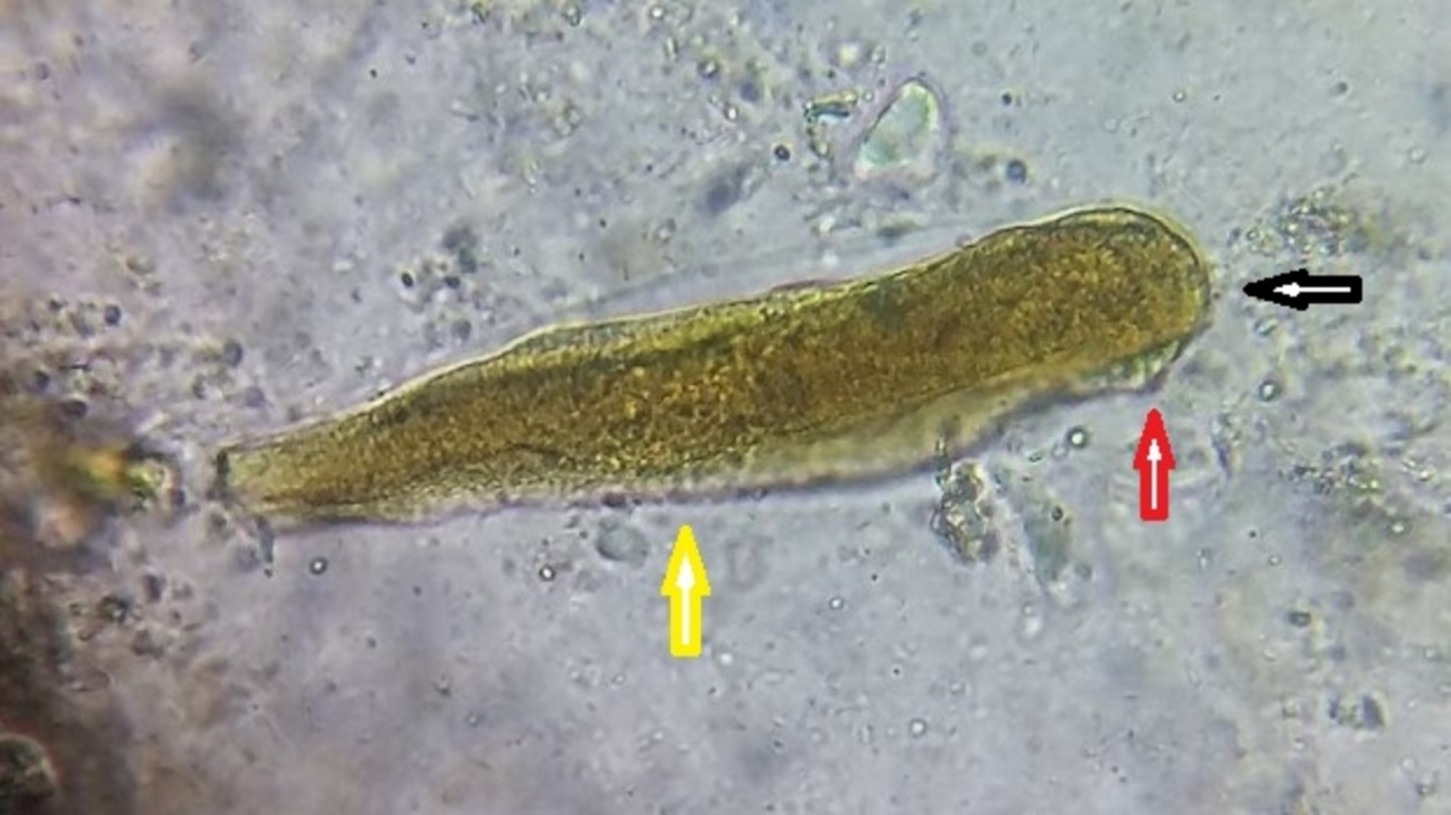
Direct stool wet mount showing the adult worm of Hymenolepis nana The adult worm revealing the rostellum (black arrow), scolex (red arrow), and broader than long segments (yellow arrow)

On an average, there were around two eggs per a high-power field (40X) indicating heavy infestation. Stool for occult blood was negative. The patient was advised a single dose of albendazole (400 mg) [4]. A repeat direct stool examination after two days of treatment revealed no eggs but showed the adult forms, as shown in Figure 2.

In view of mild polyhydramnios, the patient was advised to get admitted in the hospital for close observation. But the patient decided to leave the hospital against the physician’s advice.

## Discussion

*H*. *nana *is the most common cestode causing human infections. It is also called as *Rodentolepis nana*, *Vampirolepis nana*, *Hymenolepis fraterna*, and *Taenia nana*. It is an armed tapeworm with a retractable rostellum, a slender neck and broader than long segments, an exception with other cyclophyllidean cestodes, which have longer than broad segments. It is reported throughout the world and is more frequent in the temperate climates, the reason being the inability of the eggs to survive the harsh environmental conditions of the tropical climate. In the developing and third world countries with a tropical climate, as in India, where there is dense population, the infection appears to be frequent due to easy transmissibility from one person to the other (households, schools). Although most intestinal parasitic infections are reported among children of school-going age, there are frequent reports of adult infections including pregnant women throughout the world.

*H*. *nana *infection among pregnant women

Intestinal parasitic infections may cause severe morbidity among pregnant women, especially those living under low-socioeconomic conditions and are nutritionally deficient. Intestinal parasitic infections among pregnant women were recently reported from Colombia. This study screened 331 stool specimens and found 41% overall prevalence of parasitic infections and 9% infected with more than one parasite [5]. An Ethiopian study screened 384 stool specimens from pregnant women attending the anti-natal care center. This study found a 31.5% prevalence rate of intestinal parasitic infections with *H*. *nana* accounting for 0.3% infections [6]. A recent study from Ethiopia reported the significance of using formalin to ether sedimentation concentration method (FEC) when compared to the direct stool wet mount in the diagnosis of intestinal parasitic infections in pregnant women attending anti-natal care. This study had noted improved detection rates for intestinal parasites using FEC (24.7%) as against 18.8% when direct stool wet mount was employed (*p* < 0.001). This study had concluded that a direct stool wet mount was not a sensitive method for detecting *H*. *nana* infection in particular and other intestinal helminths [7].

A prospective study that included 14,914 pregnant women from Guatemala reported the effect of protozoal and helminthic infections on the fetal growth. This study observed that 44% were positive for parasitic infestation with 24% infected with helminths and most of the positive patients had lower nutritional status. It had concluded that intrauterine growth retardation (IUGR) was more frequent during heavy infections of protozoal and helminthic infections except in those infected with *Strongyloides stercoralis *and *H*. *nana* [8]. A study of parasitic infections and the associated complications in pregnant women from Tanzania reported that there was a 29% increased chance of anemia with helminthic infection during pregnancy [9]. A study from Ethiopia, which screened pregnant women for intestinal parasites, reported a 24.7% total prevalence of intestinal helminths with *H*. *nana* accounting for 1.6% cases. This study reported a mild to moderate risk of anemia during *H*. *nana* infection [10].

The occurrence of mixed *H*. *nana* and *H*. *diminuta *infection in a mother and her 12-year-old child was reported previously. Both patients gave a year-long history of anorexia, alternative episodes of constipation and diarrhea, abdominal pain and intestinal cramps. Also, both the patients had a good appetite and better nutritional intake [11]. This confirms the fact that the *H*. *nana* infection may be chronic and can be transmitted among households. 

Therapeutic considerations against *H*. *nana *and other helminthic infections

A previous study had reported that albendazole therapy at early childhood could not eliminate *H*. *nana* infection (2.18% and 1.19% before and after therapy) as compared to the hookworm infection (1% and 0 before and after therapy, respectively). This randomized, double‐blind, placebo‐controlled trial also highlighted the importance of prenatal treatment and its relation to allergic complications in the children born to such mothers [12].

Another study had reported that preventive chemotherapy, although eliminates the current infections cannot necessarily prevent re-infections [13]. This could be attributed to several factors including the sanitation, over-crowding, hygiene, nutrition, age, and others.

Preventive therapy with albendazole was found effective in the elimination of infection in 85% of the cases as reported from Pakistan. They found a 6% prevalence of *H*. *nana* infection among children aged between 6 and 15 years [14].

Crowded families in the impoverished rural areas could act as hotspots of intestinal helminthic infections, despite the preventive chemotherapy as observed by a recent study from Brazil [15]. This highlights the significance of overcrowding in the human–human transmission of infection and maintenance of the parasite. 

*H*. *nana *as an emerging intestinal zoonotic infection

A previous study had warranted the inclusion of hymenolepiasis as a neglected tropical parasitic disease. This prevalence study of *H*. *nana* from Angola revealed that in spite of the preventive chemotherapy, *H*. *nana* infections and its associated morbidity had not significantly reduced. It was also suggested that the prevalence of *H*. *nana* may be associated with other factors that include hygiene, sanitation, auto-infection, and co-morbidities (prevalence of other parasitic infections like schistosomiasis) [16]. A recent study impressed on the significance of hymenolepiasis as an emerging intestinal parasitic infection in children and its association with anemia [17].

Potential complications of *H*. *nana *infection

Hymenolepiasis infection turning into malignancy was reported previously, where abnormal, proliferating, and genetically altered cells, resembling the cancerous cells consisting of the *H*. *nana* DNA, were noted [18]. This signifies the importance of laboratory diagnosis and preventive chemotherapy, especially among the susceptible population.

Since most human infections by *H*. *nana* remain asymptomatic, unless there is a heavy infestation, the clinical diagnosis of infection remains elusive. This has been confirmed by a recent report of an accidental discovery of worms observed during colonoscopy in a 56-year-old patient who gave a history of working in a farm and exposure to farm animals like the livestock, dogs, and rodents [19].

The occurrence of genetically confirmed *Hymenoplepis *infection and the cross-reactivity of the parasite-borne molecules with the anti-human leukocyte antibodies was reported recently. This study impresses on the immunomodulatory mechanisms in Hymenolepiasis and other helminthic infections and the absence of anti-parasitic immune response mechanisms during human–cestode infection [20].

In the present case, it is not sure if the patient had this infection previously. The patient belonged to a low-socioeconomic group and had been continuously exposed to livestock, which could be a cause of infection. It can be hypothesized that she was actually harboring the parasite for a long time, as revealed by the presence of a huge number of eggs in the stool. Also, it is not sure if the mild polyhydramnios is a coincidence or a cause of intestinal parasitic infection. The literature clearly indicates that polyhydramnios poses an increased risk of the fetal anomalies, maternal dyspnea, pre-term labor, premature rupture of the membrane, and postpartum hemorrhage. 

## Conclusions

Intestinal parasitic infections contribute to significant morbidity among children and such infections in pregnant women pose an increased threat to fetal and maternal health. Epidemiology of intestinal parasitic infections among pregnant women has not been adequately researched. Isolated case reports of intestinal parasitic infections in a defined population, as in the case of pregnant women, should not be undermined. In view of the potential effects of parasitic infestation in the development of fetal abnormalities and maternal health, it becomes important to understand the pathophysiology of intestinal parasitic infections among pregnant women and initiate control and preventive measures.
